# A Comparative Analysis of Gene Expression Patterns and Cell Phenotypes between Cervical and Peripheral Blood Mononuclear Cells

**DOI:** 10.1371/journal.pone.0008293

**Published:** 2009-12-14

**Authors:** Rachel E. Horton, Nadine Kaefer, Elijah Songok, Fernando B. Guijon, Nadia Kettaf, Geneviève Boucher, Rafick-Pierre Sekaly, T. Blake Ball, Frank A. Plummer

**Affiliations:** 1 Department of Medical Microbiology, University of Manitoba, Winnipeg, Manitoba, Canada; 2 Kenya Medical Research Institute, Nairobi, Kenya; 3 Department of Obstetrics, Gynaecology, and Reproductive Sciences, University of Manitoba, Winnipeg, Manitoba, Canada; 4 Centre de Recherche du Centre Hospitalier de l'Université de Montréal, Montréal, Quebec, Canada; 5 National HIV and Retrovirology Laboratory, Public Health Agency of Canada, Winnipeg, Manitoba, Canada; 6 National Microbiology Laboratory, Public Health Agency of Canada, Winnipeg, Manitoba, Canada; New York University, United States of America

## Abstract

Studies of the immunological environment in the female genital tract (FGT) are critical for the development of vaccines or microbicides to halt the spread of sexually transmitted infections. Challenges arise due to the difficulties of sampling from this site, and the majority of studies have been conducted utilising peripheral blood mononuclear cells. Identifying functional differences between immune cells of the FGT and peripheral blood would aid in our understanding of mucosal immunology. We compared the gene expression profile of mononuclear cells at these two sites. Messenger RNA expression analysis was performed using gene expression arrays on matched cervical mononuclear cells and peripheral blood mononuclear cells. Further cellular phenotyping was done by 10 colour flow cytometry. Of the 22,185 genes expressed by these samples, 5345 genes were significantly differentially expressed between the cell populations. Most differences can be explained by significantly lower levels of T and B cells and higher levels of macrophages and dendritic cells in the FGT compared with peripheral blood. Several immunologically relevant pathways such as apoptosis and innate immune signalling, and a variety of cytokines and cytokine receptors were differentially expressed. This study highlights the importance of the unique immunological environment of the FGT and identifies important differences between systemic and mucosal immune compartments.

## Introduction

A major global public health issue is the large number of sexually transmitted infections (STIs), at least four of which, Human Immunodeficiency Virus (HIV), Hepatitis B Virus (HBV), Human Papilloma Virus (HPV) and Herpes simplex virus type 2 (HSV-2), remain incurable. Vaccines are available for both HBV [1] and HPV [2]; however, no vaccine is available for HIV, (with an estimated 33.2 million infections), or HSV-2 (with an estimated global prevalence of 37%). In Sub-Saharan Africa, one of the regions hardest hit by the HIV/AIDS epidemic, the primary route of HIV-1 infection is through vaginal intercourse and a disproportionately higher number of women become infected than men [3], [4]. Furthermore, not all women are capable of negotiating condom usage [5], and there are currently no effective female-controlled STI prevention methods. Therefore, there has been increased research into microbicides; however, the failure of several microbicide trials for HIV-1, including those for nonoxynol-9 and cellulose sulphate, demonstrates a fundamental lack of knowledge of the immunobiology of the female genital tract (FGT) [6], [7], [8]. A more thorough understanding of the FGT would greatly benefit research efforts concerning all STIs, but particularly HIV-1.

There is comprehensive information in the literature regarding the humoral immune response of the female genital tract [9], [10] although the role of cervical antibodies in disease prevention for a number of STIs remains controversial in part due to their low levels and therefore problematic detection [11], [12], [13]. A number of studies have examined cell mediated immune responses [14], [15], [16], [17] but there is a lack of comprehensive data on the major immune cell populations or the immune microenvironment of the FGT mucosal surface compared to the extensive studies conducted in peripheral blood. A thorough knowledge of this unique environment is key in the further development of vaccine strategies and the treatment of STIs.

Despite the paucity of information in the literature regarding cell population phenotyping or immune gene expression, several studies have documented host factors present in the FGT that play a role in susceptibility to infection, including the role of natural microbiota [18], [19], [20]. A decrease in pH, as well as a diverse array of anti-microbial peptides provides natural protection against some pathogens, but this natural barrier alone is clearly insufficient to protect against all FGT infections. The FGT is necessarily a site of immunological balance. Not only must the host be protected from pathogens, but this site must also be permissible to sperm and natural flora and specific mechanisms must be in place to allow development of the foetus. Consequently, care must be taken to ensure any alterations to immunological activity at this site do not upset this delicate balance. The use of non-invasive techniques is important for characterizing the FGT in a variety of populations for ease of sampling and allowing large enough participant numbers.

In this study we set out, for the first time, to characterise gene expression and immune cell distribution in the FGT in comparison to the systemic compartment. Two different technologies, microarray gene expression analysis and 10-colour flow cytometry, were used to obtain a more complete understanding of the cellular processes and populations and to identify novel gene expression patterns at this important site of disease transmission. These findings will provide baseline data to support future microbicide and vaccine design for numerous STIs, especially HIV-1.

## Results

### Patients

Healthy female volunteers aged 18 to 50 undergoing gynaecological exam were recruited from the Health Sciences Centre Department of Obstetrics & Gynaecology Colposcopy Clinic in Winnipeg, Manitoba. Women undergoing cervical biopsies, menstruating or pregnant were excluded from the study. The cervical mononuclear cell (CMC) sampling and isolation protocol has been previously described [20], is relatively painless and non-invasive, and is suitable for a variety of clinical settings. Ten subjects had CMC counts high enough to yield RNA of sufficient quality and concentration (≥15 ng/µl) for gene expression analysis and provided matched peripheral blood mononuclear cell (PBMC) samples as well. A further 5 subjects provided suitable CMC samples for robust flow cytometry analysis and were matched with PBMC samples from age matched controls.

### Illumina BeadChip® Gene Expression Results

To examine differences in immunobiological processes between the FGT mucosal surface and blood, CMCs and PBMCs from the same subject were compared using whole genome expression arrays. Of the 24,500 genes represented on the Illumina Human-Ref8 gene expression BeadChip array, 47% of genes (11,586) were expressed in at least one CMC or PBMC sample. Of these, 5345 genes were significantly differentially expressed between CMCs and PBMCs with a fold change of 1.3 or greater and an adjusted for multiple comparisons p-value of ≤0.05, and were compiled into a differentially expressed genes (DEG) list. 2342 genes were significantly over expressed in CMCs compared to PBMCs while the remaining 3003 were under expressed.

**Figure 1:**
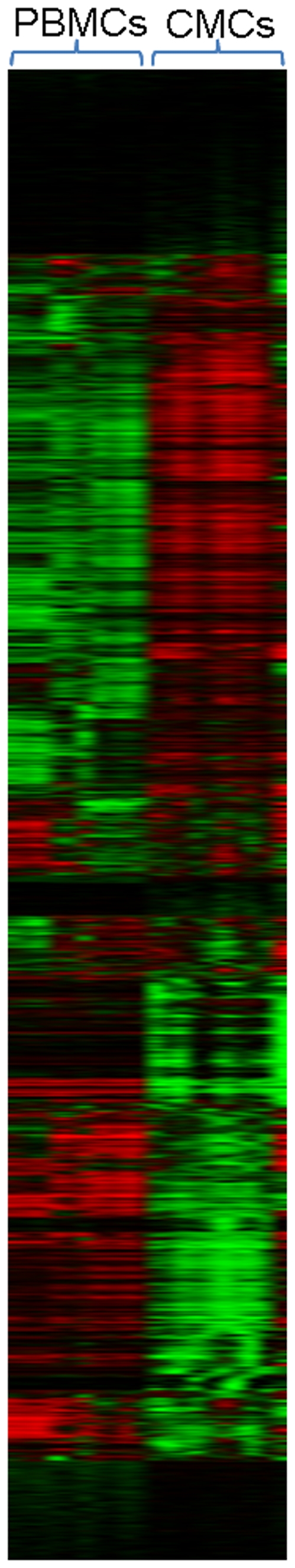
Overall gene expression in matched CMCs and PBMCs. CMCs and PBMCs cluster independently using unsupervised hierarchical clustering. Red indicates a relative increase in gene expression intensity and green represents a relative decrease in gene expression intensity in the sample.

To determine if these gene expression differences were indeed characteristic of the two cell populations, normalized gene expression intensities of all genes expressed by CMCs and/or PBMCs were inputted into Cluster® for unsupervised hierarchical clustering. CMCs and PBMCs clustered independently, as shown by Figure 1, indicating these two cell populations are genetically distinct. These data indicate CMCs and PBMCs do have unique gene expression patterns and represent distinct functional phenotypes based upon gene expression.

### 10 Colour Flow Cytometry Analysis of Cervical Versus Peripheral Blood Mononuclear Cells

**Figure 2:**
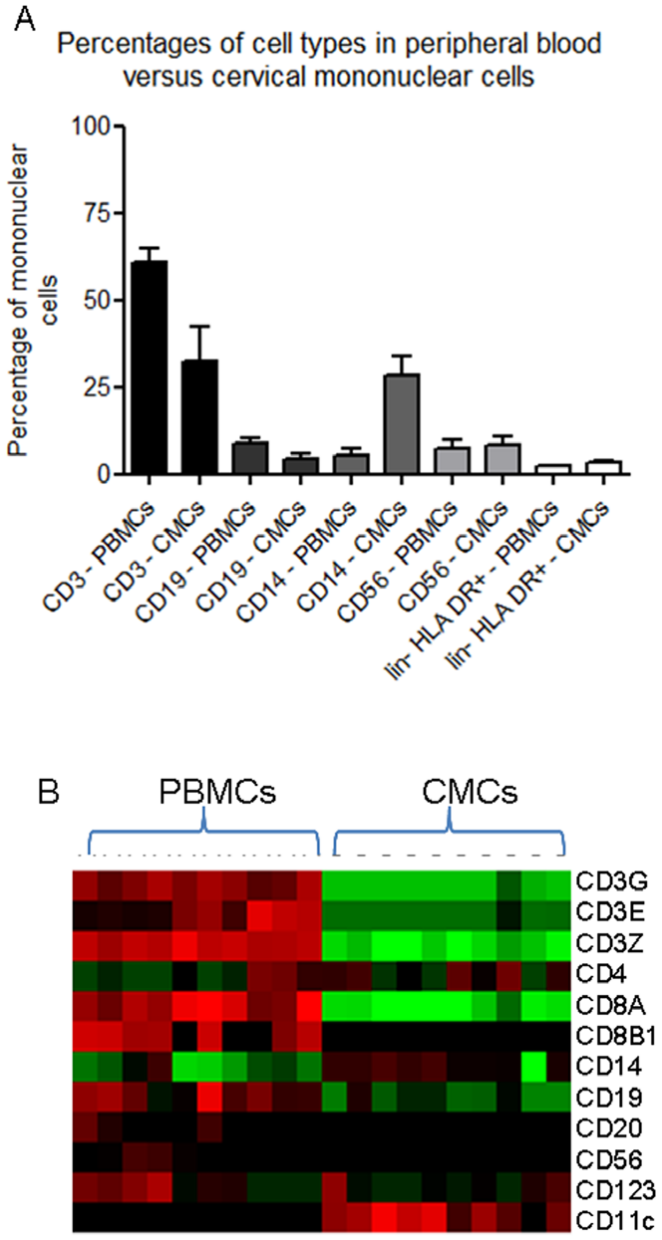
Relative differences between the immune cells of PBMC and CMC populations. (A) Columns show mean of 5 individuals with SEM shown by vertical lines. (B) Relative gene expression values of phenotyping markers. Red indicates a relative increase in gene expression intensity and green represents a relative decrease in gene expression intensity.

One of the most likely explanations for altered gene expression between CMCs and PBMCs is different cell subset makeup at these two sites, as has been described by others [16]. To understand the differences in gene expression patterns between CMCs and PBMCs, the cell population make-ups of CMCs and PBMCs were compared using 10 colour flow-cytometry and phenotyping panels focussing on antigen presenting cells. In comparison with age-matched PBMCs, CMCs showed increased proportions of CD14+ monocytes (5.5±1.9% vs 28.2±5.6%; p = 0.005) and lineage (CD3, CD14, CD19, CD56) negative, HLA-DR+ DCs (2.2±0.4% vs 3.5±0.3%; p = 0.025). CMCs showed relatively lower levels of CD3+ T cells (61.0±4.0% vs 32.3±10.0%; p = 0.028), while levels of CD56+ NK cells were the same (7.3±2.3% vs 8.3±2.3%; p = ns) and CD19+ B cells (9.0±1.4% vs 4.4±1.5%; p = 0.059) trended towards a lower level in CMCs (Fig. 2a).

These findings were confirmed by the gene expression microarray data which showed a decreased expression of T cell markers CD3 and CD8 and the B cell marker CD19 in CMCs. CD56 was equivalently expressed between PBMCs and CMCs, as was CD123 which is expressed on plasmacytoid DCs. CD14 was over expressed in CMCs, as was CD11c which is found on both myeloid DCs and monocytes, confirming the flow data (Fig. 2b). Not surprisingly, CMCs and PBMCs have divergent cell population make-ups, which critical to our understanding of immune responses at the FGT.

### Pathways Alternatively Expressed in CMCs

Although many of the differences in gene expression between CMCs and PBMCs can be explained by the observed differences in cell phenotypes present, we wanted to better understand cellular processes at the FGT. The DEG gene list (of 5345 genes) was analysed using the Database for Annotation, Visualization and Integrated Discovery (DAVID) for pathway analysis. 5011 of these GeneIDs were recognized, 1301 (26% of inputted gene list) of which were significantly enriched (p<0.05) in 25 pathways from the Kyoto Encyclopaedia of Genes and Genomes (KEGG) (Table S1). Of these, several immune regulatory pathways were identified as being differentially expressed in CMCs. Such pathways included the T cell receptor signalling pathway (48 genes), B cell receptor signalling (30 genes), natural killer cell-mediated cytotoxicity (53 genes), and the hematopoietic cell lineage pathways (37 genes) as expected based from the cell phenotyping data. Interestingly, several cellular process and metabolic pathways were also differentially regulated, including cell cycle (53 genes), apoptosis (35 genes), and ubiquitin-mediated proteolysis (51 genes), suggesting some underlying phenotypic differences between CMCs and PBMCs that may not be explained solely by makeup of the cell population.

Lists of genes identified as being over expressed or under expressed by CMCs were inputted individually to identify gene expression patterns that are over or under expressed in CMCs. This has the advantage of having smaller input gene lists so pathways over expressed or under expressed would not be lost in the noise of a larger list. Twenty-eight KEGG pathways had 653 genes (30%) significantly enriched. Pathways from the DEG list overlapped with many of these pathways; however, examining over expressed genes elucidated several novel pathways. Such pathways included the toll-like receptor signalling pathway (29 genes), complement and coagulation cascades (22 genes) and the cytokine-cytokine receptor interaction pathway (51 genes) (Table S1).

**Figure 3:**
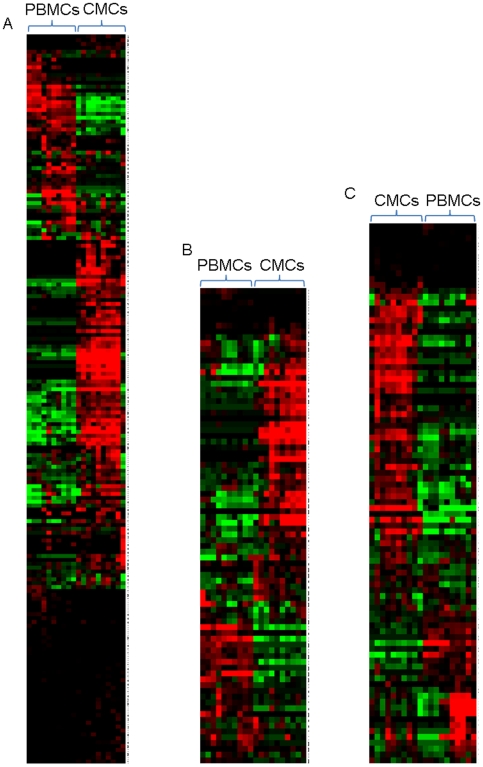
Gene expression heatmaps of differentially expressed pathways. Significant pathways were identified by DAVID with EASE scores <0.05. Red indicates a relative increase in gene expression intensity in the sample and green represents a relative decrease in gene expression intensity. Gene symbols are listed on the right of each heatmap. (A) represents all genes expressed by CMCs and PBMCs involved in the cytokine-cytokine receptor interaction pathway, (B) represents genes expressed in the apoptosis pathway, and (C) represents all genes expressed by CMCs and PBMCs involved in TLR signalling.

As immune activation plays a critical role in susceptibility to infection at the FGT, especially susceptibility to HIV-1 [21], [22], we selected apoptosis, TLR signalling and the cytokine-cytokine receptor interaction pathways for further examination due to their role in inflammation and immune activation in the FGT. Normalized fluorescence intensities of the TLR signalling, apoptosis and cytokines gene pathways (including genes not significantly differentially expressed by CMCs) also underwent unsupervised hierarchical clustering using Cluster®. Again, CMCs and PBMCs where shown to cluster independently, consistent with these main pathways being significantly differentially expressed between the two cell populations (Figure 3).

### Differentially Expressed Cytokines

Cytokines are important immune factors and thus their differential expression pattern between CMCs and PBMCs is an important difference between these immune environments. A total of 56 cytokines and cytokine receptors were over expressed in CMCs (Table S2) and there were a significantly higher number of cytokine genes in this DAVID pathway over expressed by CMCs than under expressed (22 of cytokine genes under expressed compared to 56 genes over expressed, χ^2^ = 13.14, df = 1, p = 0.0003). Figure 3a shows a heatmap of all cytokine genes expressed by CMCs and PBMCs. These over expressed cytokines include 28 well-described pro-inflammatory mediators, including MIP-1α(5.42-fold increased, p = 0.0016), MIP1β (5.52-fold up-regulated, p = 0.0035) and TNFα(3-fold upregulated, p = 5.8×10^−4^) [23], [24], and two inhibitors of inflammation; IL1R2 (2.49-fold upregulated, p = 0.001) and IL10RB (3.44-fold upregulated, p = 3.2×10^−4^) [25], [26]. Although these data do not show a significant enrichment of pro-or anti-inflammatory cytokines in the FGT over blood, they do suggest an overall increase in cytokine-mediated signalling that likely results in a more sensitive immunoregulatory inflammatory environment in the FGT.

### Apoptosis

**Figure 4:**
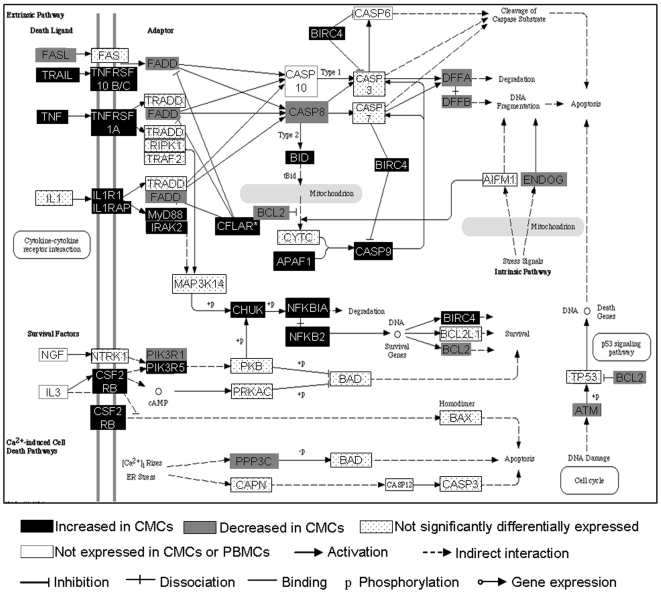
Apoptosis pathway (modified from KEGG). CFLAR, denoted by *, is an important inhibitor of apoptosis and is significantly over expressed by CMCs by a factor of 2.

Apoptosis is critically linked to inflammation and immune activation. Several genes involved in apoptosis pathways were recognised as being significantly enriched in the DEG gene list (Figure 4). Despite the apparent increase in expression of apoptosis-inducing cytokines such as TNFα (3.02-fold over expressed, p = 5.8×10^−4^), there is a concomitant up-regulation in downstream anti-apoptotic factors, including CFLAR (Caspase 8 and Fas-associated death domain-like apoptosis regulator: 2.04-fold over expressed, p = 0.0028) and X-linked inhibitor of apoptosis (XIAP: 1.73-fold over expressed, p = 1.9×10^−4^). Furthermore, genes responsible for pro-apoptotic end functions such as DNA degradation (DFFB: 1.87-fold under expressed, p = 7.63×10^−6^) and caspase cascade initiation were significantly under expressed (FADD: 1.54-fold under expressed, p = 3.9×10^−5^). Overall the data for this pathway suggests an anti-apoptotic environment in the FGT due to downstream suppression of this pathway and not as a result of a decrease in apoptosis-inducing cytokines.

### Toll-Like Receptor Signalling

**Figure 5:**
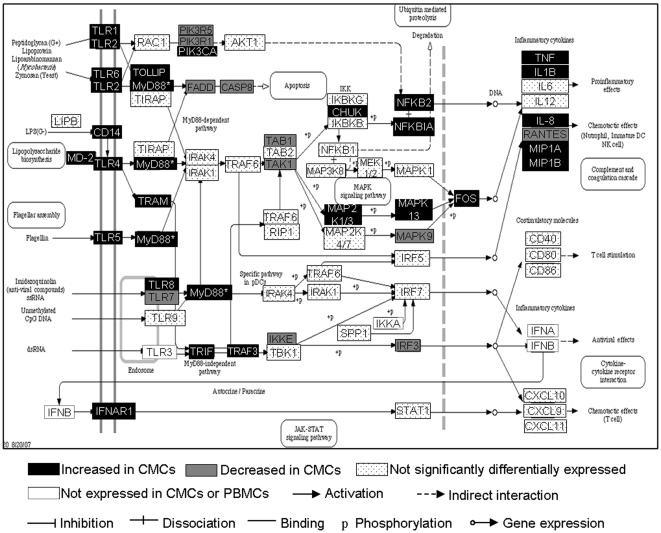
TLR signalling pathway (modified from KEGG). MYD88, denoted by *, is an important inhibitor of apoptosis and is significantly over expressed by CMCs by a factor of 2.39.

TLRs are innate signalling receptors and play an important role in the induction of an immune response at mucosal surfaces [27]. Several genes involved in the TLR pathway were significantly enriched in the CMCs upregulated gene list (Figure 5). Nearly all the TLR receptors are differentially expressed, with TLRs 1, 2, 4, 5, 6 and 8 significantly over expressed and TLR7 significantly under expressed in CMCs (p<0.05). Neither cell population expressed detectable TLR3 nor was there a significant difference in expression of TLR9. MyD88, an important regulator of the TLR signalling pathway, was significantly over expressed in CMCs 2.39-fold (p = 2.6×10^−4^) compared to PBMCs. There is also an apparent increase in some cytokine end products of TLR signalling, as demonstrated by the enrichment in cytokine gene expression, but no significant difference in costimulatory molecules CD86 or CD40 molecules, nor antiviral factors IFNα, IFNβ or IFNγ (Figure 5). Again, these differences could be due to an overrepresentation of APCs in CMCs but also suggestive of increased TLR signalling in the FGT as might be expected at a site that frequently encounters pathogenic and non-pathogenic microorganisms.

## Discussion

Research into the immune environment of the FGT is challenging and has been traditionally hampered by a number of factors, not least of all the difficulty in obtaining samples containing sufficient cell numbers for immunologic analysis. Also, due to its non-sterile nature, high levels of contamination with microorganisms as well as high mucus levels and mucosal epithelial cells complicate many types of immunologic studies. Cervical scrapings are relatively painless and non-invasive and represent one of the best options for obtaining FGT cell samples; however, there remain significant hurdles to overcome. A large number of FGT samples that have to be discarded as unsuitable. In our study, for example, around 30% of samples collected were suitable for flow cytometry while 40% were suitable for microarray which demonstrates the difficulty in obtaining sample sizes to generate good quality data. We thus utilised molecular expression array techniques and polyfunctional flow cytometry to obtain a broad understanding of immune events at this site as much as possible, so that targeted studies, utilising smaller sample sizes can be done to confirm these investigatory findings.

Based on the gene expression data, immune cells from the FGT and peripheral blood show divergent gene expression patterns. Over 5300 genes were differentially expressed between CMCs and PBMCs, and these two populations were shown to cluster independently by unsupervised hierarchical clustering analyses. The most likely reason for the majority of these differences in gene expression is the difference in cellular sub-populations of CMCs as confirmed by flow cytometry.

Several pathways indicative of specific lymphocyte subsets are differentially expressed by CMCs. B- and T-cell receptor-mediated signalling were observed to be decreased in CMCs, which can be explained by the lower number of these cell populations in the CMC samples as shown by the flow cytometry data, and is in agreement with an earlier study [16]. Prakash *et al* also demonstrated higher levels of macrophages similar to those seen in our study and in concordance with an increased requirement for innate sensing at this immunological site. Additionally, dendritic cell (DC) numbers in the FGT were increased. Presumably the high levels of foreign antigen at this mucosal surface give rise to an increased requirement for antigen presenting cells and immune surveillance. However, several important immunological pathways that may be common to the cervical cell population were identified by the gene expression analyses that would not have been identified by cell phenotyping alone. This is important and demonstrates the strength of these techniques as it provides information as to cell physiology and function, not just cell phenotype.

Three particularly important immunoregulatory pathways were differentially expressed in CMCs: cytokine-cytokine receptor interactions, apoptosis, and toll-like receptor signalling. There is an increase in the expression level of cytokine genes in immune cells from the FGT when compared with those from peripheral blood. We did not find a bias toward up-regulation of pro-inflammatory genes in CMCs compared to PBMCs, (22 cytokines down-regulated by CMCs: 20 pro-inflammatory and two anti-inflammatory), but we did however see an overall increase in cytokine gene expression (56 were up regulated). This global increase in primary inflammatory cytokine signalling suggests a tightly regulated cytokine-cytokine receptor environment within the FGT. However, several factors are known to regulate cytokine expression. Of several factors known to regulated cytokine expression, both apoptosis [28] and TLR signalling [27], [29] were found to be differentially regulated in CMCs in this study.

Despite the increased expression of apoptosis-inducing molecules by CMCs, such as TNF and TRAIL, there is what appears to be an overall inhibition of apoptosis pathways, suggesting CMCs may be more resistant to the induction of apoptosis. CFLAR is a well-described inhibitor of the extrinsic pathway, and XIAP plays a role in suppressing the intrinsic apoptotic pathway [30], [31]. The extrinsic apoptotic pathway plays an important role in immune regulation. Inducing apoptosis in neutrophils, for example, causes suppression of the transcription of pro-inflammatory cytokines, leading to the induction of an anti-inflammatory response in macrophages and to an immunosuppressive state [28]. The up-regulation of CFLAR, as seen in this study, coupled with the down-regulation of genes responsible for DNA degradation, suggests a state present in the FGT [32] that would promote cellular survival and may, in part, explain the increased pro-inflammatory cytokine expression observed in CMCs. The intrinsic apoptosis pathway is potentially inhibited by the up-regulation of XIAP. This suppression of apoptosis also suggests a state of increased basal immune activation in the FGT over the systemic immune compartment. Further functional studies will be necessary to confirm these findings.

TLRs are an important component of innate immune surveillance [29], and increased innate immune signalling in the FGT has been reviewed elsewhere [33]. The data in this study supports an increase in TLR expression and signalling, complete with the induction of inflammatory cytokines in the absence of active infection. What is most striking about the observed TLR expression in the FGT is the apparent biased increase in TLRs recognising bacterial pathogenic patterns: TLRs 1, 2, 4, 5, 6 and 8, despite the presence of normal flora [27]. Accordingly, there is an increase in neutrophil chemotactic molecules (such as MIP-1α/β) and no change in interferon gene expression. The close proximity of vaginal natural flora to this immune microenvironment could possibly lead to bacterially-induced TLR signalling and the apparent inflammatory state. These results may also be a result of increased antigen presenting cells in the CMC population, but highlight the importance of immune sensing at the FGT. Further investigations concerning the interplay between the vaginal microbiome and TLR signalling in the endocervix would be interesting in order to further elucidate the immune microenvironment of the FGT.

This study is one of the first to examine the immune cell population makeup in conjunction with genome wide gene expression analyses in the FGT of healthy donors. The data presented in this paper shows a unique gene expression patterns in CMCs compared to PBMCs. This difference in gene expression is partially explained by differences in cell populations, with significantly elevated macrophage and lineage-negative DCs and reduced T cell and B cell populations. Thus there is skewing towards higher frequencies of myeloid and lower frequencies of lymphoid-derived leukocytes being present in the FGT compared with the periphery. This study also demonstrates that, in addition to the differences in cellular subpopulations, CMCs and PBMCs differentially express cytokines, apoptosis, and the TLR signalling pathway, three factors that regulate inflammation and possibly suggest a chronic inflammatory state present in the genital tract. This increased, chronic level of inflammation is an important to understand in the context of susceptibility to STIs, especially viruses such as HIV-1 that show increased transmission at inflammatory sites [34]. This study shows that the immunologic environment of the FGT is significantly different from that of the circulatory system. Understanding these differences is important for understanding mucosal immune responses to pathogens and ultimately for designing future microbicide or mucosal vaccine candidates.

## Materials and Methods

### Ethics Statement

This study was conducted according to the principles expressed in the Declaration of Helsinki. All studies were approved by the University of Manitoba Health Research Ethics Board. All patients provided written informed consent for the collection of samples and subsequent analysis.

### Study Scrapings and Samples

Cervical samples were obtained from healthy female participants from the Health Sciences Centre Department of Obstetrics & Gynaecology Colposcopy Clinic in Winnipeg, Manitoba undergoing routine cervical smears. All women were of European descent and between the ages of 18 and 50. Any women menstruating, pregnant or presenting with an STI at the time of sampling were excluded from the study. For gene expression analysis, matched peripheral blood was obtained by venipuncture from the same donors at the time of examination. For 10-colour flow cytometry analysis, peripheral blood was obtained from age-matched female lab volunteers. All studies were approved by the University of Manitoba Health Research Ethics Board.

PBMCs were isolated from heparinized whole blood by density gradient centrifugation using ficoll–hypaque (Bio-Lynx, Gibco) and washed twice in PBS (Sigma) containing 10% FBS (Gibco). CMCs were separated from cervical epithelial cells by this same method. Cells were counted and viability assessed using Trypan Blue (Sigma-Aldrich). CMC samples with less than 50% cell viability, or visible blood or epithelial cell contamination were discarded. 10 matched CMC and PBMC samples were used for gene expression analyses.

Flow cytometry using CMCs is notoriously challenging due to low cell numbers and high background fluorescence. A high number of samples were unusable for data analysis (approx 75%), leaving 5 suitable CMC samples and their age matched PBMCs.

### 10 Colour Flow Cytometry

**Figure 6:**
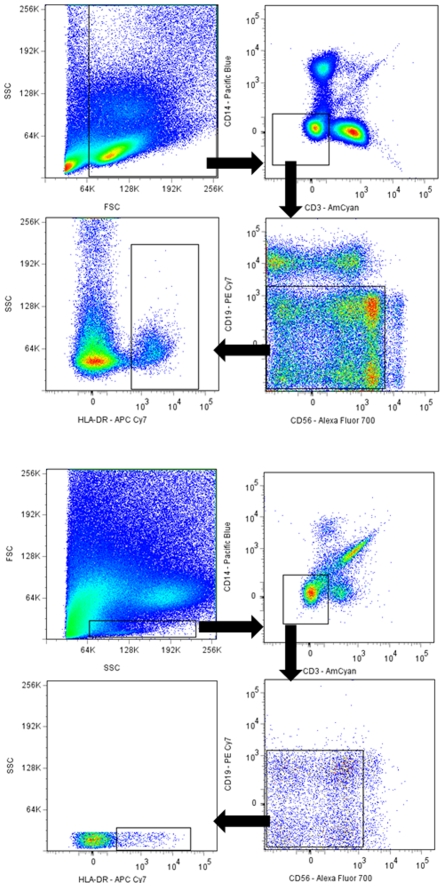
Gating scheme. Representative dot plots showing the gating scheme used to determine different mononuclear cell subtypes and via lineage negative gating obtain the Dendritic cell population of PBMCs (A) and CMCs (B). Cells were obtained from peripheral blood and cervical scrapings by ficoll separation and labelled with a cocktail of antibodies (CD3-AmCyan, CD14-Pacific Blue, CD19-PECy7, CD56-Alexa Fluor 700 and HLA DR- APCCy7) then 10 colour flow cytometry was performed on a LSR II.

PBMCs and CMCs were suspended in RPMI and aliquoted into tubes at approximately 10^6^ cells per tube. Cells were then incubated with fluorochrome-conjugated antibodies (CD3-AmCyan, CD14-Pacific Blue, CD19-PECy7, CD56-Alexa Fluor 700 and HLA DR-APCCy7 [BD]). After 1 hr, 4°C in the dark the cells were washed in PBS-2% FCS and resuspended in 1% paraformaldehyde (Sigma). Samples were analyzed using a LSR II flow cytometer (Becton Dickenson) and primarily gated by forward and side scatter profile. For both CMCs and PBMCs the area to be gated was determined after preliminary experiments using back-gating to determine the location of the lymphocyte populations and 10,000 events were collected through this gate. As there is no one DC specific marker, as has been described previously [35], DCs were gated by negative selection for CD3, CD14, CD19, CD56 and positive selection for HLA-DR as shown in Figure 6. Lineage negative populations were also counted in the process.

### RNA Isolation and Quality Assessment

Total RNA was extracted using TrIzol® (Invitrogen) according to the reagent-enclosed protocol. RNA was then DNase-digested using Qiagen RNase-free DNase Set and purified of contaminating organics using the Qiagen RNeasy MinElute Kit. RNA concentration and quality was assessed on an Agilent BioAnalyzer 2100 RNA Nano 6000 Series II according to manufacturer's instructions.

### Microarray Analysis

150 ng of total RNA was amplified and labelled using the Ambion Illumina® RNA Amplification kit according to manufacturer's instructions. 750 ng of cRNA was hybridized onto Illumina® Human-Ref8 v2 Expression BeadChip arrays as previously described [36].

### Microarray Data Analysis

Illumina probe data were exported from BeadStudio as raw data and screened for quality. Raw signal intensities were pre-processed in R (www.r-project.org). As such, we filtered out probes with intensity below background in all samples. Average background intensities were calculated from designated background and address only beads. We then used this mean background value to surrogate beads with intensity below this average. Data was then quantile normalized and log2 transformed. Fold changes were calculated by dividing raw CMC gene signal intensities by PBMC values and paired p-values were calculated and adjusted with Benjamini-Hochberg False Discovery Rate [37] in LIMMA. Microarray data is MIAME compliant, and raw and normalized data have been submitted to the ArrayExpress online database, accession number E-MTAB-143. Genes with an absolute fold change of ≥1.3 and a p-value≤0.05 were considered significantly differentially expressed. Significant genes were analysed for immune pathway enrichment by DAVID. Samples were hierarchically clustered with an uncentred Pearson correlation using Cluster® and visualised with TreeView® (EisenLab) [38].

### Statistics

Data analyses of flow cytometry data were carried out using Prism version 3.0 (GraphPad Software) using the unpaired t-test to compare different cell populations. Comparison of the proportion of pro- and anti-inflammatory cytokines expressed was also performed in this program using a Chi-square test.

## Supporting Information

Table S1 All pathways differentially expressed by CMCs
(0.07 MB DOC)

Table S2 All differentially expressed cytokines
(0.11 MB DOC)
