# Supplementary material for: A Comparative Analysis of Gene Expression Patterns and Cell Phenotypes between Cervical and Peripheral Blood Mononuclear Cells
Source: PLoS One. 2009 Dec 14;4(12):e8293. doi: 10.1371/journal.pone.0008293 (PMC2790076; doi:10.1371/journal.pone.0008293)
Supplement: Table S1 — All pathways differentially expressed by CMCs (0.07 MB DOC) [file pone.0008293.s001.doc]

**Table S1. All pathways differentially expressed by CMCs**

| **Pathway** | **List^a^** | **Pop^b^** | **p** | **Gene list** |
| --- | --- | --- | --- | --- |
| T cell receptor signalling pathway | 48 | 94 | 6.85E-05 | all_diff_exp^c^ |
| Ribosome | 50 | 100 | 9.32E-05 | all_diff_exp |
| Epithelial cell signalling in *H. pylori* infection | 37 | 68 | 1.09E-04 | all_diff_exp |
| Cholera - Infection | 25 | 41 | 2.08E-04 | all_diff_exp |
| Alzheimer's disease | 19 | 28 | 2.59E-04 | all_diff_exp |
| Cell cycle | 53 | 114 | 5.54E-04 | all_diff_exp |
| Oxidative phosphorylation | 57 | 128 | 0.00121 | all_diff_exp |
| Galactose metabolism | 18 | 32 | 0.00696 | all_diff_exp |
| B cell receptor signalling pathway | 30 | 65 | 0.0128 | all_diff_exp |
| NK cell mediated cytotoxicity | 53 | 129 | 0.0130 | all_diff_exp |
| Hematopoietic cell lineage | 37 | 85 | 0.0155 | all_diff_exp |
| Pyrimidine metabolism | 37 | 88 | 0.0277 | all_diff_exp |
| N-Glycan biosynthesis | 20 | 42 | 0.0342 | all_diff_exp |
| Apoptosis | 35 | 84 | 0.0373 | all_diff_exp |
| Ubiquitin mediated proteolysis | 51 | 131 | 0.0422 | all_diff_exp |
| Insulin signalling pathway | 52 | 134 | 0.0426 | all_diff_exp |
| Aminoacyl-tRNA biosynthesis | 18 | 38 | 0.0490 | all_diff_exp |
| Complement/coagulation cascades | 22 | 69 | 0.00119 | CMC_up^d^ |
| TLR signalling pathway | 29 | 102 | 0.00122 | CMC_up |
| Galactose metabolism | 12 | 32 | 0.00642 | CMC_up |
| Aminosugars metabolism | 11 | 28 | 0.00682 | CMC_up |
| Adipocytokine signalling pathway | 19 | 73 | 0.0263 | CMC_up |
| Huntington's disease | 10 | 29 | 0.0264 | CMC_up |
| Cytokines and cytokine receptors | 51 | 256 | 0.0418 | CMC_up |
| Fructose and mannose metabolism | 12 | 42 | 0.0498 | CMC_up |
| Purine metabolism | 38 | 145 | 9.24E-04 | CMC_down^e^ |
| RNA polymerase | 11 | 23 | 0.00121 | CMC_down |
| Folate biosynthesis | 14 | 41 | 0.00667 | CMC_down |
| DNA polymerase | 10 | 24 | 0.00692 | CMC_down |
| Pyruvate metabolism | 14 | 42 | 0.00834 | CMC_down |
| Parkinson's disease | 9 | 21 | 0.00967 | CMC_down |
| Citrate cycle | 10 | 30 | 0.0322 | CMC_down |
| Aminophosphonate metabolism | 7 | 17 | 0.0357 | CMC_down |
| Phe, Tyr and Trp biosynthesis | 5 | 9 | 0.0371 | CMC_down |
| Histidine metabolism | 12 | 41 | 0.0417 | CMC_down |
| Proteasome | 8 | 22 | 0.0417 | CMC_down |

^a^Number of genes from inputted list involved in the pathway

^b^Number of genes in the population involved in the pathway. Total gene population: 4214

^c^Total number of genes from gene list recognized by DAVID: 1301

^d^Total number of genes from gene list recognized by DAVID: 653. CMC_up and CMC_down to do not include pathways previously discovered by all_diff_exp

^e^Total number of genes from gene list recognized by DAVID: 651
