# Supplementary material for: A Comparative Analysis of Gene Expression Patterns and Cell Phenotypes between Cervical and Peripheral Blood Mononuclear Cells
Source: PLoS One. 2009 Dec 14;4(12):e8293. doi: 10.1371/journal.pone.0008293 (PMC2790076; doi:10.1371/journal.pone.0008293)
Supplement: Table S2 — All differentially expressed cytokines (0.11 MB DOC) [file pone.0008293.s002.doc]

**Table S2**: All differentially expressed cytokines

| **Gene symbol** | **Fold change** | **Regulation** | **p-value** |
| --- | --- | --- | --- |
| IL8RB | 20.83 | Up | 5.9E-05 |
| OSM | 18.00 | Up | 4.0E-04 |
| IL8RA | 15.97 | Up | 9.8E-05 |
| CCL20 | 13.40 | Up | 1.0E-03 |
| CSF3R | 12.71 | Up | 8.7E-05 |
| CXCL6 | 9.10 | Up | 2.3E-05 |
| IL1R2 | 9.05 | Up | 3.8E-05 |
| TNFSF14 | 7.82 | Up | 9.1E-06 |
| CXCL2 | 6.55 | Up | 4.2E-04 |
| TNFRSF1A | 6.14 | Up | 7.7E-06 |
| TNFRSF21 | 5.89 | Up | 1.4E-05 |
| LTBR | 5.50 | Up | 2.5E-06 |
| CCL3 | 5.42 | Up | 1.6E-03 |
| IL1RAP | 4.18 | Up | 6.5E-05 |
| CXCL1 | 4.02 | Up | 1.9E-02 |
| CSF2RA | 3.77 | Up | 3.7E-06 |
| CCR1 | 3.77 | Up | 9.8E-06 |
| IL1RAP | 3.71 | Up | 9.8E-05 |
| TNFRSF10B | 3.45 | Up | 8.9E-04 |
| IL10RB | 3.44 | Up | 3.2E-04 |
| TNFSF14 | 3.24 | Up | 7.6E-05 |
| IFNAR1 | 3.21 | Up | 7.0E-04 |
| TNFRSF8 | 3.16 | Up | 2.9E-04 |
| TNF | 3.02 | Up | 5.8E-04 |
| IL6R | 2.96 | Up | 1.0E-04 |
| PLEKHQ1 | 2.83 | Up | 1.6E-04 |
| CSF2RB | 2.58 | Up | 4.1E-03 |
| IL19 | 2.56 | Up | 5.8E-03 |
| CCL23 | 2.55 | Up | 1.1E-02 |
| IL13RA1 | 2.49 | Up | 2.7E-05 |
| IL1R2 | 2.49 | Up | 1.0E-03 |
| VEGF | 2.49 | Up | 3.4E-03 |
| TNFRSF1B | 2.48 | Up | 6.9E-03 |
| TNFSF10 | 2.48 | Up | 1.0E-02 |
| IL19 | 2.47 | Up | 7.3E-03 |
| IL1R1 | 2.37 | Up | 9.9E-05 |
| IFNGR2 | 2.37 | Up | 3.2E-04 |
| TNFRSF12A | 2.36 | Up | 6.1E-03 |
| CXCL16 | 2.33 | Up | 3.9E-03 |
| IFNGR1 | 2.22 | Up | 1.2E-03 |
| IL17R | 2.09 | Up | 2.0E-03 |
| ACVR1B | 2.04 | Up | 2.0E-03 |
| IL6R | 2.01 | Up | 9.3E-04 |
| IL8 | 1.95 | Up | 1.3E-03 |
| CCL18 | 1.92 | Up | 1.9E-02 |
| TNFRSF6B | 1.89 | Up | 1.9E-04 |
| TNFSF15 | 1.82 | Up | 1.3E-02 |
| TNFRSF9 | 1.82 | Up | 8.2E-03 |
| CCR3 | 1.78 | Up | 7.6E-03 |
| TNFRSF10C | 1.69 | Up | 1.1E-02 |
| CX3CL1 | 1.63 | Up | 3.6E-02 |
| TNFSF9 | 1.63 | Up | 4.4E-03 |
| LIF | 1.54 | Up | 2.0E-02 |
| MET | 1.51 | Up | 2.9E-02 |
| LEP | 1.48 | Up | 3.4E-02 |
| TNFRSF13B | 1.31 | Down | 1.4E-02 |
| BMPR2 | 1.32 | Down | 2.3E-02 |
| FASLG | 1.49 | Down | 2.9E-02 |
| IL12RB1 | 1.61 | Down | 7.6E-04 |
| MPL | 1.64 | Down | 4.4E-02 |
| IL21R | 1.84 | Down | 1.8E-03 |
| FLT3LG | 1.90 | Down | 1.4E-03 |
| CXCR6 | 1.98 | Down | 7.0E-03 |
| IL10RA | 2.14 | Down | 2.1E-03 |
| TGFBR2 | 2.37 | Down | 2.8E-05 |
| IL15 | 2.37 | Down | 3.8E-05 |
| IL7R | 2.72 | Down | 6.5E-04 |
| TNFRSF7 | 3.60 | Down | 6.3E-05 |
| LTB | 3.77 | Down | 4.1E-05 |
| CCL5 | 4.77 | Down | 2.3E-04 |
| CCL8 | 5.39 | Down | 4.2E-02 |
| CXCR3 | 5.59 | Down | 1.0E-04 |
| IL2RB | 5.64 | Down | 2.7E-05 |
| IL11RA | 6.50 | Down | 9.2E-06 |
| PPBP | 6.81 | Down | 1.7E-03 |
| CCR7 | 7.46 | Down | 2.7E-06 |
| TNFRSF25 | 11.22 | Down | 2.1E-06 |
